# Prolonged infusion of bivalirudin after elective percutaneous coronary intervention protects against procedural myocardial injury (a COBER study)—a randomized trial

**DOI:** 10.1038/s41598-023-34008-y

**Published:** 2023-04-24

**Authors:** Zhiming Wu, Peina Meng, Yajie Guo, Wei You, Xiangqi Wu, Fei Ye

**Affiliations:** Department of Cardiology, Nanjing First Hospital, Nanjing Medical University, 68 Changle Rd, Nanjing, 210006 China

**Keywords:** Cardiology, Interventional cardiology

## Abstract

Procedural myocardial injury (PMI), which is the most common complication of elective percutaneous coronary intervention (ePCI), is associated with future adverse cardiac events. In this randomized pilot trial, we assessed the effects of prolonged use of the anti-coagulant bivalirudin on PMI after ePCI. Patients undergoing ePCI were randomized into the following two groups: the bivalirudin use during operation group (BUDO, 0.75 mg/kg bolus plus 1.75 mg/kg/h) and the bivalirudin use during and after operation for 4 h (BUDAO, 0.75 mg/kg bolus plus 1.75 mg/kg/h). Blood samples were collected before and 24 h after ePCI (per 8 h). The primary outcome, PMI, was defined as an increase in post-ePCI cardiac troponin I (cTnI) levels of > 1 × 99th% upper reference limit (URL) when the pre-PCI cTnI was normal or a rise in cTnI of > 20% of the baseline value when it was above the 99th percentile URL, but it was stable or falling. Major PMI (MPMI) was defined as a post-ePCI cTnI increase of > 5 × 99th% URL. A total of 330 patients were included (*n* = 165 per group). The incidences of PMI and MPMI were not significantly higher in the BUDO group than in the BUDAO group (PMI: 115 [69.70%] vs. 102 [61.82%], *P* = 0.164; MPMI: 81 [49.09%] vs. 70 [42.42%], *P* = 0.269). However, the absolute change in cTnI levels (calculated as the peak value 24 h post-PCI minus the pre-PCI value) was notably larger in the BUDO group (0.13 [0.03, 1.95]) than in the BUDAO group (0.07 [0.01, 0.61]) (*P* = 0.045). Moreover, the incidence of bleeding events was similar between the two groups (BUDO: 0 [0.00%]; BUDAO: 2 [1.21%], *P* = 0.498). Prolonged infusion of bivalirudin for 4 h after ePCI reduces PMI severity without increasing the risk of bleeding.

ClinicalTrials.gov.Number: NCT04120961, 09/10/2019.

## Introduction

ePCI is considered as a safe approach to treating chronic coronary syndrome (CCS), with a very low incidence of major procedural complications^1,2^. However, the levels of cardiac biomarkers, especially those of high-sensitivity cardiac troponin (hs-cTn), are frequently increased after ePCI^3^. PMI is defined as stated in the Fourth Universal Definition of Myocardial Infarction (UDMI) (post-PCI cTn elevation of > 1 × 99th percentile URL) and has been associated with a worse prognosis^3–5^. Although different therapeutic strategies have been developed to reduce PMI after PCI, the high incidence highlights the need for new therapeutic concepts^6^.

Bivalirudin is a naturally occurring oligopeptide analog of hirudin that reversibly binds to the active site and the external site I of thrombin. Bivalirudin effectively inhibits free fibrin-bound thrombin, is not inactivated by platelet factor 4, does not require antithrombin to exert its activity, does not bind to proteins and matrices other than thrombin, has low immunogenicity, and does not cause liver-induced thrombocytopenia^7,8^. Compared with unfractionated heparin (UN), the important theoretical advantage of bivalirudin lies in the lack of platelet activation and rebound effect characterized by increased thrombin activity and myocardial infarction^9^. Several clinical studies showed that bivalirudin reduces the bleeding rate without loss of the antithrombotic effect^8,9^. Bivalirudin is widely used in the preoperative and intraoperative anticoagulant treatment of patients with acute coronary syndrome (ACS) after PCI, and is increasingly applied in the intraoperative anticoagulant treatment during ePCI^8–11^.

The pathophysiological mechanisms of PMI during ePCI are not fully understood, and activation of platelets and inflammation, along with distal embolization and microvascular obstruction, seem to play an important role^5^. The mechanical stress produced by PCI can release particulate debris, thrombotic and soluble substances into coronary circulation. Physical substances block coronary microcirculation, while soluble substances induce endothelial dysfunction and promote vasoconstriction. Coronary microvascular occlusion and dysfunction lead to plaque micro-infarction, accompanied by inflammatory reactions, both of which lead to PMI^12^. In autopsy analysis of sudden death patients without overt myocardial infarction, microemboli were found in microcirculation and characterized by atherosclerotic plaque materials, including cholesterol crystals, hyalin and platelet aggregates, which was also identified in the microemboli removed from the protective device in PCI patients^13,14^. The iatrogenic plaque rupture is the trauma of atherosclerotic coronary artery lesions caused by the PCI procedure. The release of plaque materials lead to microcirculation dysfunction, and its severity ranges from asymptomatic to partial or total microvascular occlusion, namely the so-called slow or non-flow phenomenon. This is an often unexpected complication in 0.5–1% of patients, with a poor clinical prognosis^15–18^.

In the Intracoronary Stenting and Antithrombotic Regimen: Rapid Early Action for Coronary Treatment (ISAR-REACT) 3 trial, 30-day outcomes in 4,570 biomarker-negative patients undergoing PCI after clopidogrel loading revealed less bleeding with bivalirudin compared with UN, but no difference in the 30-day net clinical benefit was observed^19^. Additionally, it has been reported that the rate of total bleeding event was not significantly higher with a post-PCI bivalirudin infusion for 4 h than without post-PCI infusion^8,9^. In the present randomized pilot study, we evaluated whether prolonged use of bivalirudin for 4 h after ePCI has a cardioprotective effect on PMI in patients undergoing ePCI.

## Methods

### Study participants

This randomized, open-label, parallel group design study was conducted in our hospital and was registered at ClinicalTrials.gov (Registry No., NCT04120961, 09/10/2019). The study protocol was approved by the Ethics Committee of our hospital (Approval No., KY20190823-05). All procedures complied with the principles laid down in the Declaration of Helsinki, and all participants provided written informed consent. 330 patients enrolled between September 2019 and July 2022 were randomly assigned to one of two groups: the treatment with bivalirudin during operation (control) group (n = 165) and the treatment with bivalirudin during and after operation for 4 h (intervention) group (n = 165). The computer-generated random numbers corresponding to the related group was concealed in an envelope, and was kept by an independent assistant until the treatment was assigned. All investigators were blinded to the randomization sequence. After obtaining written consents from eligible subjects, the independent assistant opened the envelope containing the allocation information. Bivalirudin was administered as an intravenous bolus of 0.75 mg/kg, followed by an infusion of 1.75 mg/kg/h.

Consecutive patients aged 18–100 years with de novo coronary lesions, ePCI, and only a single coronary artery treated at this time were considered for enrollment. The following patients were excluded: those who met the diagnostic criteria of acute myocardial infarction, patients with cardiogenic shock, patients with multiple organ failure, patients allergic to contrast agent, patients who do not tolerate dual antiplatelet therapy, patients who do not tolerate anticoagulation agents, recently infected patients, patients with hepatorenal dysfunction, thrombotic lesions of the coronary artery, and/or chronic total coronary occlusion lesion, patients with complex coronary bifurcation requiring a two-stent strategy, patients with severe coronary calcified lesions, patients with percutaneous coronary angioplasty, patients with directional coronary atherectomy or rotational atherectomy, patients undergoing drug-coated balloon treatment, patients with a bioabsorbable vascular scaffold implantation, patients who previously underwent percutaneous coronary intervention, patients with a coronary artery bypass graft, or patients with active stage of autoimmune disease.

### Study procedure and relevant medications

During hospitalization, demographic, clinical, biochemical, and echocardiographic data, as well as medication use, were recorded. All patients were given aspirin (100 mg per day) and clopidogrel (75 mg per day) or ticagrelor (90 mg twice per day) routinely, and a loading dose of clopidogrel (300 mg) or ticagrelor (180 mg) was prescribed 6–24 h before coronary angiography (CAG) and PCI. During PCI, a bolus of bivalirudin (0.75 mg/kg) was administered after insertion of the arterial sheath, followed immediately by an infusion of 1.75 mg/kg/h until completion of the PCI. Bivalirudin was then stopped at the end of PCI or prolonged in accordance with the treatment group. In addition, PCI-related data were recorded. After PCI, all patients were advised to take aspirin (100 mg per day) for life and clopidogrel (75 mg per day) or ticagrelor (90 mg twice per day) for at least 12 months according to current guidelines^20,21^.

After injection of 200-μg nitroglycerin into the coronary artery, using the guiding catheter for calibration, the end-diastolic lesion length (LL), minimum luminal diameter (MLD), reference vessel diameter (RVD), and percentage of diameter stenosis (DS) were measured before and after ePCI. CAG was performed in all patients, and CAG data were analyzed by an experienced technician blinded to the patient groups.

### Study endpoints

Primary endpoints were PMI or MPMI, and secondary endpoints were in-hospital major adverse cardiac events (MACEs) or bleeding events.

PMI was defined as an increase in cTnI levels of > 1 × 99th percentile URL in patients with a normal baseline value (≤ 99th percentile URL) or an increase in cTnI levels of > 20% of the baseline value when it was above the 99th percentile URL but it was stable or falling. MPMI was defined as an elevation of cTnI levels of more than five times the 99th percentile URL^6,22^. Creatine kinase-MB (CK-MB) and cTnI levels were analyzed at admission, before PCI, and at 8, 16, and 24 h after ePCI.

MACEs were defined as cardiovascular-related death, acute myocardial infarction, or repeated target lesion revascularization.

According to the Bleeding Academic Research Consortium (BARC) classification system^23^, bleeding events were graded from 0 to 5 as follows: 0, no bleeding or bruising; 1, bleeding (including bruising), which did not require the patient to seek medical care; 2, overt bleeding events requiring medical care (but not level 3 events); 3, bleeding requiring blood transfusion or hospitalization; 4, bleeding related to coronary artery bypass grafting; 5, fatal bleeding.

### Statistical analysis

According to a previous report^5^, the rate of PMI in patients undergoing ePCI is about 68.0% according to cTn levels. We hypothesized that the rate of PMI after prolonged bivalirudin use for 4 h after ePCI in the intervention group would be about 34.0% in the light of our pretested results. Based on this hypothesis, using a 5% significance level with a two‐sided Z test, we calculated that the minimum required sample size to achieve a power of 1.00 was 300 participants using PASS11.0 software (NCSS, LLC). Assuming 10% of participants would be lost to follow-up, the total sample size of this study was 330.

Normally distributed continuous variables are expressed as mean ± standard deviation; non-normally distributed continuous variables are expressed as median with interquartile range (IQR); and categorical variables are expressed as percentages. The Kolmogorov–Smirnov test was used to evaluate the distributions of continuous variables. Normally distributed continuous variables were compared by the Student *t*-test. Non-normally distributed data were compared using the Mann–Whitney *U* test. Categorical variables were compared using the χ^2^ test. In all statistical analysis, a two-tailed *P*-value of < 0.05 was considered as statistically significant. All statistical analyses were conducted using SPSS 24.0 (SPSS Inc, Chicago, IL, USA).

### Ethics approval

The ethics committee of Nanjing First Hospital approved the study protocol (Approval No., KY20190823-05), and all methods were performed in accordance with relevant guidelines and regulations. Additionally, written informed consent was obtained from all patients before cardiac catheterization.

## Results

### Baseline clinical characteristics in the BUDO and BUDAO groups

**Figure 1:**
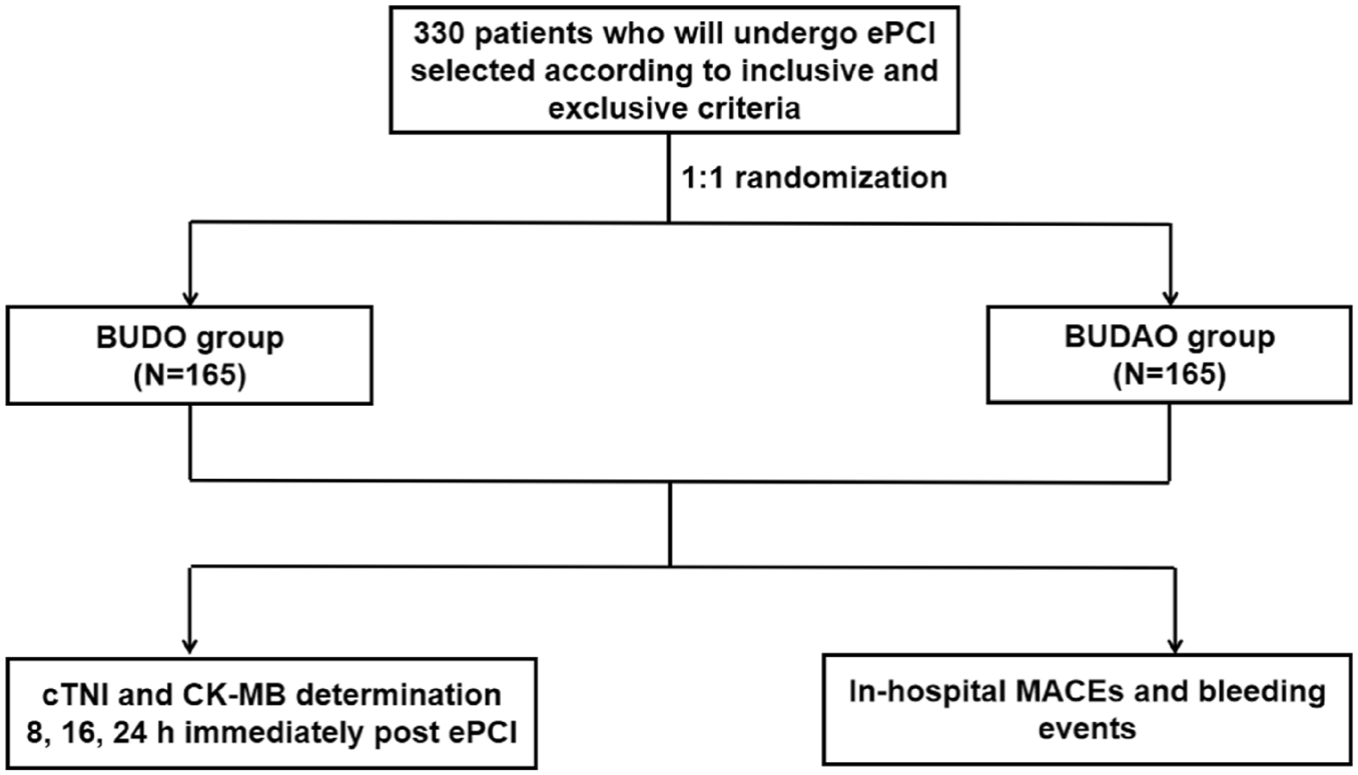
Flowchart of this study. ePCI, Elective percutaneous coronary intervention; cTnI, Cardiac troponin I; CK-MB, Creatine kinase-MB.

The flow chart is shown in Fig. 1. For each group, 330 participants who were randomly assigned, received intended treatment, and were analysed for the primary outcome. No significant differences in age, gender, height, body weight, cardiovascular risk factors (hyperlipidemia, hypertension, diabetes mellitus, smoking, and renal insufficiency), or previous drug therapy (anti-platelet medication, angiotensin-converting enzyme inhibitors/angiotensin receptor blockers [ACEIs/ARBs], β-blockers, calcium channel blockers [CCB], and statins) were detected between the BUDO and BUDAO groups (*P* > 0.05) (Table 1).

**Table 1:** Basic clinical characteristics in the BUDO and BUDAO groups.

Variables	BUDO group (n = 165)	BUDAO group (n = 165)	*P* value
Age (years)	65.63 ± 11.22	66.35 ± 10.80	0.552
Male gender (%)	115 (69.70%)	119 (72.12%)	0.716
Height (cm)	165.98 ± 7.93	166.07 ± 7.23	0.913
Body weight (kg)	68.76 ± 11.98	67.92 ± 11.30	0.512
Hyperlipidemia (%)	118 (71.52%)	114 (69.09%)	0.718
Hypertension (%)	129 (78.18%)	118 (71.52%)	0.204
Diabetes mellitus (%)	50 (30.30%)	62 (37.58%)	0.201
Current smoker (%)	71 (43.03%)	75(45.45%)	0.740
Renal insufficiency (%)	15 (9.09%)	12 (7.27%)	0.689
Biochemical indexes			
WBC (^10^9^)	6.52 ± 1.55	6.46 ± 1.46	0.709
RBC (^10^12^)	4.34 ± 0.56	4.25 ± 0.60	0.182
Hb (g/L)	132.43 ± 16.98	129.93 ± 16.41	0.175
BG (mmol/L)	6.31 ± 1.90	6.39 ± 2.02	0.700
BUA (μmol/L)	359.41 ± 132.18	348.99 ± 123.63	0.460
TG (mmol/L)	1.70 ± 0.94	1.68 ± 0.99	0.854
TC (mmol/L)	4.07 ± 1.14	4.04 ± 1.21	0.834
HDL-C (mmol/L)	1.00 ± 0.25	0.96 ± 0.23	0.184
LDL-C (mmol/L)	2.31 ± 0.93	2.33 ± 1.00	0.871
PLT (^10^9^)	193.83 ± 61.74	200.47 ± 57.05	0.311
APTT (sec)	27.92 ± 3.35	28.30 ± 3.38	0.299
TT (sec)	18.45 ± 9.07	18.30 ± 4.50	0.845
FIB (g/L)	3.32 ± 1.07	3.43 ± 1.08	0.319
Previous drug therapy			
Anti-platelet medication (%)	81 (49.09%)	73 (44.24%)	0.440
ACEI/ARB (%)	94 (56.97%)	108 (65.45%)	0.142
β-blocker (%)	86 (52.12%)	98 (59.39%)	0.223
CCB (%)	58 (35.15%)	52 (31.52%)	0.817
Statin (%)	89 (53.94%)	80 (48.48%)	0.378
Echocardiographic parameters			
AOD (mm)	32.97 ± 3.27	32.55 ± 3.37	0.247
IVSD (mm)	10.32 ± 1.77	10.56 ± 2.34	0.288
LVDD (mm)	49.21 ± 5.40	49.27 ± 5.83	0.922
LVPWD (mm)	9.49 ± 1.35	9.48 ± 1.18	0.931
LVDS (mm)	32.73 ± 6.14	33.04 ± 6.68	0.668
LVEF (%)	59.51 ± 9.12	58.92 ± 9.55	0.568

Data were expressed as n (%), mean ± SD. *BUDO* Bivalirudin use during operation; *BUDAO* Bivalirudin use during and after operation; *WBC* White blood cell; *RBC* Red blood cell; *Hb* Hemoglobin; *BG* Blood glucose; *BUA* Blood urine acid; *TG* Triglyceride; *TC* Total cholesterol; *HDL-C* High-density lipoprotein cholesterol; *LDL-C* Low-density lipoprotein cholesterol; *PLT* Platelet; *APTT* Activated partial thromboplastin time; *TT* Thrombin time; *FIB* Fibrinogen; *ACEI*/*ARB* Angiotensin-converting enzyme inhibitors/angiotensin receptor blockers; *CCB* calcium channel blockers; *AOD* Aortic diameter; *IVSD* Interventricular septum diameter; *LVDD* Left ventricular enddiastolic diameter; *LVPWD* Left ventricular posterior wall diameter; *LVDS* Left ventricular endsystolic diameter; *LVEF* Left ventricular ejection fraction.

Furthermore, no significant differences in biochemical indices (white blood cell [WBC], red blood cell [RBC], hemoglobin [Hb], blood glucose [BG], blood urine acid [BUA], triglyceride [TG], total cholesterol [TC], high-density lipoprotein cholesterol [HDL-C], low-density lipoprotein cholesterol [LDL-C], platelet [PLT], activated partial thromboplastin time [APTT], thrombin time [TT], and fibrinogen [FIB]) and parameters (aortic diameter [AD], interventricular septal diameter [IVSD], left ventricular end-diastolic diameter [LVDD], left ventricular posterior wall diameter [LVPWD], left ventricular end-systolic diameter [LVDS], and left ventricular eject fraction [LVEF]) were observed between the BUDO and BUDAO groups (*P* > 0.05) (Table 1).

These results show that baseline clinical data were similar between the two groups.

### Coronary angiography results and PCI procedural data in the BUDO and BUDAO groups

Target vessel revascularization (TVR) (left anterior descending artery [LAD], left circumflex artery [LCX], or right coronary artery [RCA]), LL, MLD, distal RVD, and DS before ePCI and in-stent MLD, distal RVD, and DS after ePCI in the BUDO group were similar to those in the BUDAO group (*P* > 0.05) (Table 2).

**Table 2:** Coronary angiography results before and after PCI in the BUDO and BUDAO groups.

Variables	BUDO group (n = 165)	BUDAO group (n = 165)	*P* value
Target vessel			0.337
LAD (%)	97 (58.79%)	86 (52.12%)	
LCX (%)	23 (13.94%)	32 (19.39%)	
RCA (%)	45 (27.27%)	47 (28.48%)	
Before PCI			
LL (mm)	36.99 ± 20.58	34.56 ± 18.39	0.260
MLD (mm)	0.76 ± 0.31	0.74 ± 0.25	0.519
Distal RVD (mm)	2.73 ± 0.43	2.70 ± 0.39	0.519
DS (%)	72.16 ± 10.86	72.87 ± 7.56	0.487
After PCI			
In-stent MLD (mm)	2.36 ± 0.43	2.32 ± 0.40	0.473
Distal RVD (mm)	2.75 ± 0.42	2.73 ± 0.40	0.656
DS (%)	14.54 ± 6.76	15.09 ± 6.10	0.440

Data were expressed as n (%), mean ± SD. *PCI* Percutaneous coronary intervention; *LAD* Left anterior descending artery; *LCX* Left circumflex artery; *RCA* Right coronary artery; *LL* Lesion length; *MLD* Minimal lumen diameter; *RVD* Reference vessel diameter; *DS* Diameter stenosis.

During lesion preparation, the maximal diameter (MD), the length and pre-dilation maximal pressure of the pre-dilation balloon (PRDB), the pre-dilation number, and pre-dilation total time (TT) were not significantly different between the BUDO and BUDAO groups (*P* > 0.05). During stent implantation, the number, average diameter (AD), total length (TL), deployed maximal pressure (DMP), and deployed total time (DTT) of stents in the BUDO group were very similar to those in the BUDAO group (*P* > 0.05). Finally, during the post-dilation period, no statistical differences in MD, length, and post-dilation maximal pressure (POMP) of the post-dilation balloon (PODB), post-dilation number, and post-dilation TT were observed between the two groups (*P* > 0.05). Additionally, the utilization rate of intravascular ultrasound (IVUS), intra-aortic balloon pump (IABP), verapamil, and tirofiban, the incidence of no/slow blood flow and operation time were not significantly different between the BUDO and BUDAO groups (*P* > 0.05) during the ePCI period (Table 3).

**Table 3:** PCI procedural data and incidence of PMI in the BUDO and BUDAO groups.

Variables	BUDO group (n = 165)	BUDAO group (n = 165)	*P* value
Pre-dilation			
PRDB MD (mm)	2.57 ± 0.32	2.53 ± 0.36	0.214
PRDB length (mm)	13.93 ± 2.82	14.27 ± 3.11	0.293
PRDB PRMP (atm)	13.32 ± 2.69	13.80 ± 3.19	0.143
Pre-diated number	3.00 ± 1.05	2.85 ± 0.81	0.145
Pre-diated TT (s)	9.98 ± 3.60	9.62 ± 2.82	0.317
Stent implantation			
Stent number	1.59 ± 0.72	1.48 ± 0.64	0.148
Stent AD (mm)	3.07 ± 0.47	3.02 ± 0.42	0.256
Stent TL (mm)	40.81 ± 21.49	38.38 ± 18.79	0.276
Stent DMP (atm)	10.13 ± 1.99	9.98 ± 1.67	0.474
Stent DTT (s)	6.36 ± 2.94	6.42 ± 2.29	0.835
Post-dilation			
PODB MD (mm)	3.25 ± 0.49	3.16 ± 0.48	0.110
PODB length (mm)	12.12 ± 2.22	12.19 ± 2.26	0.769
PODB POMP (atm)	18.80 ± 2.43	18.56 ± 2.53	0.376
Post-dilated number	3.64 ± 1.05	3.45 ± 1.20	0.121
Post-dilated TT (s)	12.27 ± 3.46	11.78 ± 3.31	0.190
During PCI			
IVUS use (%)	24 (14.55%)	20 (12.12%)	0.627
IABP use (%)	2 (1.21%)	0 (0.00%)	0.498
No/slow blood flow (%)	2 (1.21%)	4 (2.42%)	0.685
Verapamil use (%)	1 (0.61%)	4 (2.42%)	0.371
Tirofiban use (%)	1 (0.61%)	0 (0.00%)	1.000
Operation time (min)	40.52 ± 24.72	43.22 ± 23.76	0.312
After PCI			
PMI (%)	115 (69.70%)	102 (61.82%)	0.164
MPMI (%)	81 (49.09%)	70 (42.42%)	0.269

Data were expressed as n (%), mean ± SD. *PMI* Procedural myocardial injury; *PRDB* Pre-dilated balloon; *MD* Maximal diameter; *PRMP* Pre-dilated maximal pressure; *TT* Total time; *AD* Average diameter; *TL* Total length; *DMP* Deployed maximal pressure; *DTT* Deployed total time; *PODB* Post-dilated balloon; *POMP* Post-dilated maximal pressure; *IVUS* Intravascular ultrasound; *IABP* Intra-aortic balloon pump; *MPMI* Major PMI.

These results reveal that CAG results and PCI procedural data were very similar between the two groups.

### Levels of myocardial biomarkers before and after ePCI, in-hospital MACEs, and bleeding events in the BUDO and BUDAO groups

**Figure 2:**
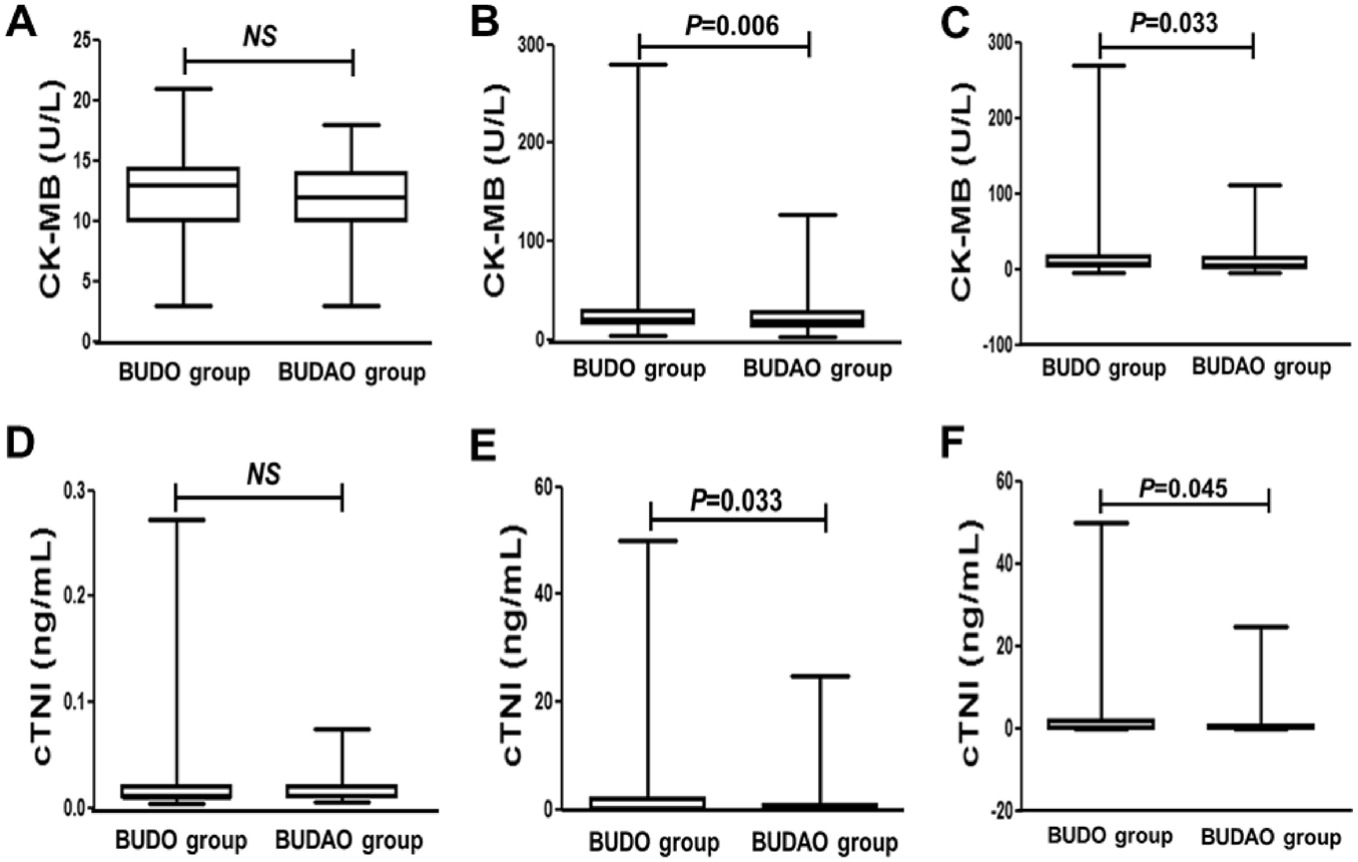
Levels of myocardial biomarkers before and after ePCI in the BUDO and BUDAO groups. (**A**,**B**) CK-MB levels between two groups before and after PCI; (**C**). CK-MB changes between two groups before and after PCI; (**D**,**E**). cTNI levels between two groups before and after PCI; (**F**) cTNI changes between two groups before and after PCI. NS, Not significance. Data were expressed as median (Q1, Q3).

Before ePCI, CK-MB and cTnI levels were similar in the BUDO and BUDAO groups (*P* > 0.05) (Fig. 2A,D). However, after ePCI, these indices were significantly higher in the BUDO group than in the BUDAO group (*P* < 0.05 or *P* < 0.01) (Fig. 2B,E). Furthermore, differences between pre- and post-ePCI values for these two indices were significantly larger in the BUDO group than in the BUDAO group (*P* < 0.05) (Fig. 2C,F). No significant differences in the incidence of PMI and MPMI were observed between the BUDO and BUDAO groups (*P* > 0.05) (Table 3).

The incidence of in-hospital MACEs, including target vessel (TV) acute myocardial infarction (AMI), TVR, and TV cardiac death (CD), was not significantly higher in the BUDO group compared to the BUDAO group (*P* > 0.05). Additionally, after prolonged use of bivalirudin in the BUDAO group, the incidence of bleeding events according to BARC criteria was not significantly increased compared to the BUDO group (*P* > 0.05) (Table 4).

**Table 4:** In-hospital MACEs and bleeding events in the BUDO and BUDAO groups.

Variables	BUDO group (n = 165)	BUDAO group (n = 165)	*P* value
MACEs (%)	1 (0.60%)	0 (0.00%)	1.000
TV-AMI (%)	1 (0.60%)	0 (0.00%)	
TVR (%)	0 (0.00%)	0 (0.00%)	
TV-CD (%)	1 (0.60%)	0 (0.00%)	
Bleeding events (BARC grading)	0 (0.00%)	2 (1.21%)	0.498
1 (%)	0 (0.00%)	0 (0.00%)	
2 (%)	0 (0.00%)	1 (0.60%)	
3 (%)	0 (0.00%)	1 (0.60%)	
4 (%)	0 (0.00%)	0 (0.00%)	
5 (%)	0 (0.00%)	0 (0.00%)	

Data were expressed as n (%). *MACEs* Major adverse cardiac events; *TV-AMI* Target vessel-acute myocardial infarction; *TVR* Target vessel revasculization; *TV-CD* Target vessel-cardiac death; *BARC* Bleeding academic research consortium.

These results indicate that prolonged use of bivalirudin after ePCI could reduce PMI in patients undergoing ePCI without increasing the risk of bleeding.

## Discussion

A multicenter, randomized, double-blind, placebo-controlled trial (The ISAR-REACT 3 trial) has shown that although bivalirudin treatment significantly reduced the incidence of in-hospital bleeding, it did not provide a net clinical benefit (defined as the quadruple endpoint of death, myocardial infarction, TVR, or in-hospital major bleeding) at 30 days in myocardial biomarker-negative patients with stable and unstable coronary artery disease undergoing PCI after pre-treatment with 600-mg clopidogrel^19^. Gargiulo et al. reported that in ACS patients with or without ST-segment elevation, the primary endpoint (a composite of urgent TVR, definite ST, or net adverse clinical events) did not differ between groups with or without post-PCI bivalirudin infusion, but a post-PCI full dose (1.75 mg/kg/h for ≤ 4 h) at the operator’s discretion was associated with improved outcomes when compared with no or low-dose post-PCI infusion (0.25 mg/kg/h for at least 6 h) or heparin^24^. However, currently, no research data are available that indicate whether infusion of a full dose of bivalirudin for 4 h after ePCI has a protective effect on PMI in patients compared with infusion of a full dose of bivalirudin during ePCI. CK and its isoenzyme CK-MB have always been the preferred biomarkers for the diagnosis of myocardial infarction before the appearance of cTn^25^. After acute MI, CK-MB levels begin to increase at 2–4 h, reach a peak at 12 h, and recover to baseline levels within 48–72 h^26^. It has been acknowledged that cTn is a more sensitive and specific cardiac biomarker than CK-MB^5,26^. After the onset of acute MI, cTn levels begin to rise at 3–6 h and reach a peak at 10–24 h; cTnT levels return to baseline values within 10–15 days, and cTnI levels take 5–7 days^5,26,27^. In this study, we chose cTnI and CK-MB serum levels to determine whether PMI occurred and its severity.

In the present study, we found that infusion of a full dose of bivalirudin after ePCI for 4 h exerted a cardioprotective effect, as indicated by significantly reduced levels of cardiac biomarkers (CK-MB and cTnI), even though the incidence of PMI was not markedly decreased compared with bivalirudin use during operation. Furthermore, prolonged use of bivalirudin did not increase the risk of bleeding. It is reported that a post-PCI full dose of bivalirudin (1.75 mg/kg/h for ≤ 4 h) could reduce the incidence of myocardial infarction or major bleeding when compared with no or low-dose post-PCI infusion or heparin in ACS patients with or without ST-segment elevation^24^. Totally, these results indicated that prolonged use of bivalirudin at the full dose (1.75 mg/kg/h) after PCI had a cardio-protective effect in patients with or without ACS.

In the observational study of Sheikh-Taha et al., using bivalirudin as a bolus followed by infusion for the duration of the intervention did not significantly reduce the incidence of the primary endpoints (a composite of death, acute MI, or an urgent need for TVR during hospitalization) but significantly increased bleeding events in patients undergoing ePCI compared with the bolus only group^28^. First, the design of their study was different from ours. Second, changes in levels of cardiac biomarkers (CK-MB and cTnI) between two groups were not implied in this study, and therefore the advantage of prolonged bivalirudin use lied in the alleviation of PMI as indicated by myocardial biomarkers in our study, even if the incidence of PMI was not significantly influenced in both studies. Finally, prolonged use of bivalirudin did not significantly increase the risk of bleeding in both studies. The key factors that influence the incidence and magnitude of PMI could be broadly classified into patient factors (such as age and renal insufficiency), CAG-related factors (such as LL and MLD), and procedural factors (such as pre-dilation and post-dilation procedures)^6^. In our study, baseline clinical data, CAG results, and PCI-related procedural data were similar between the two groups, indicating that these PMI-related factors had a small impact on the results of our study.

During plaque rupture resulting from PCI, the coronary arterial endothelial barrier is damaged, and connective tissue elements, atherosclerotic materials, and subendothelial matrix proteins (von Willebrand factor, collagen) are exposed and released into the blood. Platelets adhere to collagen and von Willebrand factor through specific cell receptors (glycoprotein [GP] Ia/IIa, GP Ib-IX, GP VI) and then become activated. Clotting factors, chemotaxins, and activated platelets de-granulate and secrete agonists and vasoconstrictors, which can promote thrombin generation, platelet aggregation, and vasospasm. Eventually, these pathophysiological changes lead to coronary microvascular circulation disorder (capillary obstruction, impaired vasomotion, pletelet-leukocyte aggregation, coronary microembolization, capillary rupture and hemorrhage), which is closely associated with PMI^6,29,30^. The coronary microvascular circulation is also a victim of myocardial ischemia and reperfusion^29,30^. Thrombin plays an important role in coagulation and platelet activation during or after PCI^7^. In these settings, direct inhibition of thrombin is an attractive treatment strategy. Bivalirudin, a polypeptide hirudin analog of 20 amino acids, binds reversibly and bivalently with the thrombin molecule to inhibit its action^7,31^. In the present study, we found that prolonged use of bivalirudin after ePCI had a cardioprotective effect on PMI, as indicated by significantly reduced myocardial biomarkers without increased incidences of blood bleeding events, revealing that intensive thrombin inhibition could improve microcirculatory disorder, which is closely related to PMI development in patients with ePCI.

### Limitations

This is a single-center clinical study; our results must be verified in multicenter studies. We found that bivalirudin after ePCI had a tendency to reduce the incidence of PMI, and the reason was that there is a relative small myocardial injury in ePCI patients^32^. Therefore, we need to increase the sample size for further investigations. In the context of the fact that vasoactive soluble factors released during plaque rupture are involved in the development of PMI^12^, the limitation of intensive bivalirudin therapy presented here. Graft atherosclerosis of patients with chronic kidney disease is more plaque calcification and necrosis, but less vasoconstrictor potential^33^. Prolonged use of bivalirudin after ePCI might increase the financial burden of patients.

### Conclusion

In conclusion, prolonged infusion of bivalirudin after ePCI reduced the severity of PMI, as indicated by reduced serum CK-MB and cTnI levels. Furthermore, it did not significantly increase the incidence of in-hospital bleeding events.

## Data Availability

The data that support the findings of this study are available from the corresponding author upon reasonable request.
